# Unhealthy Ultra-Processed Food Consumption in Children and Adolescents Living in the Mediterranean Area: The DELICIOUS Project

**DOI:** 10.3389/ijph.2025.1608318

**Published:** 2025-10-21

**Authors:** Alice Rosi, Francesca Giampieri, Osama Abdelkarim, Mohamed Aly, Achraf Ammar, Evelyn Frias-Toral, Juancho Pons, Laura Vázquez-Araújo, Alessandro Scuderi, Nunzia Decembrino, Alice Leonardi, Fernando Maniega Legarda, Lorenzo Monasta, Ana Mata, Adrián Chacón, Pablo Busó, Giuseppe Grosso

**Affiliations:** ^1^ Human Nutrition Unit, Department of Food and Drug, University of Parma, Parma, Italy; ^2^ Department of Clinical Sciences, Università Politecnica delle Marche, Ancona, Italy; ^3^ Research Group on Food, Nutritional Biochemistry and Health, Universidad Europea del Atlántico, Santander, Spain; ^4^ International Joint Research Laboratory of Intelligent Agriculture and Agri-Products Processing, Jiangsu University, Zhenjiang, China; ^5^ Faculty of Sport Sciences, Assiut University, Assiut, Egypt; ^6^ ESLSCA University Egypt, Giza, Egypt; ^7^ Department of Training and Movement Science, Institute of Sport Science, Johannes Gutenberg-University Mainz, Mainz, Germany; ^8^ Research Laboratory, Molecular Bases of Human Pathology, LR19ES13, Faculty of Medicine, University of Sfax, Sfax, Tunisia; ^9^ School of Medicine, Universidad Espíritu Santo - Samborondón, Guayaquil, Ecuador; ^10^ Editorial Luis Vives (EDELVIVES), Carretera de Madrid, Zaragoza, Spain; ^11^ BCC Innovation, Technology Center in Gastronomy, Basque Culinary Center, Donostia-San Sebastián, Spain; ^12^ Basque Culinary Center, Faculty of Gastronomic Sciences, Mondragon Unibertsitatea, Donostia-San Sebastián, Spain; ^13^ Department of Agriculture, Food and Environment, University of Catania, Catania, Italy; ^14^ Neonatal Intensive Care Unit, AOU Policlinico G. Rodolico San Marco, Catania, Italy; ^15^ Department of Biomedical and Biotechnological Sciences, University of Catania, Catania, Italy; ^16^ Universidade Internacional do Cuanza, Cuito, Bié, Angola; ^17^ Universidad de La Romana, La Romana, Dominican Republic; ^18^ Institute for Maternal and Child Health – IRCCS Burlo Garofolo, Trieste, Italy; ^19^ Technological Institute for Children’s Products & Leisure AIJU, Alicante, Spain; ^20^ Center for Human Nutrition and Mediterranean Foods (NUTREA), University of Catania, Catania, Italy

**Keywords:** ultra-processed food (UPF), children and adolescents, mediterranean area, eating habits, lifestyle behaviours

## Abstract

**Objectives:**

This study addressed the consumption of ultra-processed foods (UPFs) formulated with excess of energy/fats/sugars (hence deemed as unhealthy) and factors associated with it in children and adolescents living in 5 Mediterranean countries participating to the DELICIOUS (UnDErstanding consumer food choices & promotion of healthy and sustainable Mediterranean diet and LIfestyle in Children and adolescents through behavIOUral change actionS) project.

**Methods:**

A total of 2011 parents of children and adolescents (6–17 years) participated in a survey exploring their children’s frequency consumption of unhealthy UPFs and demographic, eating, and lifestyle habits.

**Results:**

Most children consumed unhealthy UPFs daily: higher intake was associated with being older and with obesity, as well as higher parental education and younger age. Children eating more frequently out of home and with a higher number of meals were also more likely to consume unhealthier UPF. Moreover, more screen time and a lower healthy lifestyle score were associated with higher unhealthy UPF consumption.

**Conclusion:**

consumption of unhealthy UPFs seems to be preeminent in children and adolescents living in the Mediterranean area and associated with an overall unhealthy lifestyle.

## Introduction

Healthy eating is essential during every stage of growth and throughout life, with childhood playing an important timing for cognitive development and general health [1]. Numerous factors are involved in children’s and adolescents’ food preferences and eating behaviors [2]. These preferences continually change over time as they are affected by biological, environmental, and social factors [3]. Dietary choices are influenced by individual preferences accompanied by the evolution of food markets and the growing availability of industrial food products, which determine both the economic convenience and sensorial properties [4]. The so-called “nutrition transition” phenomenon results in a progressive abandonment of traditional dietary patterns in favor of “Westernized” food products characterized by increased energy and lower nutritional density [5]. Recent research involving Mediterranean countries has revealed that a large share of children and adolescents are likely to have poor adherence to traditional dietary patterns, such as the Mediterranean Diet [6], eventually preferring diets richer in foods that have undergone various industrial transformations able to improve their palatability [5].

In recent years, the level of food processing has been the focus of major attention due to its potentially adverse effects on human health [7]. According to the Nova classification, ultra-processed foods (UPFs) can be defined as industrially manufactured products containing little to no whole foods and characterized by cosmetic alterations and additives that increase sensorial properties [8]. Enhanced palatability is a common feature of most industrialized food products, including carbonated drinks and fast foods, among others [9]. Besides, enhanced palatability is not obtained at no cost, with rather important changes in sodium, added sugar, and unhealthy fat content, often accompanied by reduction in dietary fibre, protein, vitamins, and minerals, which may explain, from a mechanistic point of view, their observed detrimental effects on human health [8]. While the role of processing and additives remains unclear and not fully elucidated, the scarce nutritional quality of certain foods is known to impact one’s health [10, 11]. Many studies emphasize the inadequate nutritional quality of certain UPFs and indicate that consuming them in large quantities can increase the risk of several health issues, such as metabolic disorders, overweight, and type-2 diabetes, resulting in higher risk of cardiovascular diseases [12], kidney disease and hepatic steatosis [13]. Furthermore, recent research has pointed out that consuming UPFs may be linked to a range of serious health risks beyond those typically recognized. In particular, individuals who regularly include these foods in their diets may face a heightened likelihood of developing mental health issues, such as depression [14]. Additionally, there is evidence suggesting an increased susceptibility to gastrointestinal disorders like irritable bowel syndrome [15]. Moreover, adolescents consuming high amounts of UPF might be at a greater risk for respiratory conditions, including asthma and wheezing, which can significantly impact their quality of life [14, 16]. Regarding the impact of UPFs on the health of children and adolescents, some studies have found that frequent consumption of UPFs was associated with food addiction in overweight children [17], and also, in several prospective studies, higher consumption of UPFs during childhood has been associated with a faster increase in body mass index (BMI), body fat percentage, and waist circumference in adolescence and early adulthood [18, 19]. In this context, identifying the characteristics associated with higher consumption of unhealthy UPFs might be of interest to better target children and adolescents for educational intervention studies. Several studies have been previously conducted [20, 21], but only a minority provide information concerning Mediterranean countries. The aim of this study was to investigate the consumption of unhealthy UPF in children and adolescents living in 5 Mediterranean countries participating to the DELICIOUS project (“Understanding consumer food choices & promotion of healthy and sustainable Mediterranean diet and Lifestyle in Children and adolescents through behavIOUral change actionS”) [22].

## Methods

### Study Population

This study is a cross-sectional analysis carried out as part of the DELICIOUS project involving a survey targeting parents of children and adolescents aged 6 to 17 from five Mediterranean countries: Italy, Spain, Portugal, Egypt, and Lebanon. Participants were recruited on a voluntary basis after invitation via a consumer database previously established by one of the study partners. The aim was to gather a minimum of 400 participants from each country. Based on recent literature aiming to the same purposes in Mediterranean countries [23–25], a target of about 400 individuals per each Mediterranean country was set. The data collection was assessed via an electronic survey and a total of 2,011 individuals were finally recruited. All procedures followed the guidelines of the World Medical Association’s Declaration of Helsinki (1989), and every participant provided informed consent prior to involvement in the study.

### Data Collection

Data on participants’ demographic characteristics and lifestyle was collected. For parents, information on sex, age, education level, and occupation were recorded, while for children/adolescents, sex, age, and anthropometric measurements were noted. The children’s ages were divided into four categories: 6–8 years, 9–11 years, 12–14 years, and 15–17 years. Parental education was categorized into three levels: low (primary school), medium (secondary school), and high (tertiary education). Employment status was classified as unemployed or currently employed. The BMI of the children/adolescents was calculated based on their weight and height and classified according to the percentile ranges of the Centers for Disease Control and Prevention (CDC) growth charts for children and adolescents aged 2–19 years [26]. Participants were categorized as normal weight (BMI 5th-84th percentile), overweight (BMI 85th-94th percentile), and obese (BMI ≥95th percentile). The quality of lifestyle was assessed using the Electronic Kids Dietary Index (E-KINDEX) [27], which includes three main areas: food group intake (13 items), food beliefs and behaviors (8 items), and eating practices (9 items). For this study, only the lifestyle domains were considered. Physical activity levels were measured using the International Physical Activity Questionnaire-Short Form (IPAQ) [28], which collects information on physical activities (walking, moderate and vigorous intensity activities) over the past 7 days, including weekly frequency and daily duration of each activity. Physical activity levels were classified as low, moderate, and high according to IPAQ guidelines. Lastly, sleep duration was categorized according to National Sleep Foundation recommendations [29] into three groups: less than 8 h, 8–10 h, and more than 10 h. Screen time was divided into less than 2 h per day, 2–4 h per day, and more than 4 h per day.

### Dietary Assessment

The consumption of UPF was assessed through a food frequency questionnaire (FFQ) comprising 13 questions investigating the intake of known unhealthy food groups from group 4 of the Nova classification, such as sweets and candies, salty snacks, fast food, soft drinks, commercial sauces, etc. For this study, the median frequency consumption of all items was calculated, and participants were categorized as frequent consumers if they fell into the upper median consumption range, or daily consumers if at least one item was reported to be consumed with daily frequency.

### Statistical Analysis

Categorical variables are presented as frequencies and percentages, with the Chi-square test used to assess differences between groups of UPF consumption. Continuous variables are expressed as means and standard deviations (SDs), with the ANOVA test used to examine differences between groups. Multivariate logistic regression models were applied to calculate odds ratios (ORs) and 95% confidence intervals (CIs) for variables potentially associated with UPF consumption. All reported P-values were based on two-sided tests and compared to a significance level of 5%. SPSS 21 software (SPSS Inc., Chicago, IL, USA) was used for all statistical calculations.

## Results

The median unhealthy UPF consumption in the study sample was 1.8 servings/day, while 95% of children and adolescents resulted in daily consumption of unhealthy UPFs. The main demographic characteristics of the parents and children/adolescents enrolled in the study according to the consumption of UPF are presented in Table 1. A higher proportion of children/adolescents overweight and with obesity (p < 0.001) among those consuming more UPF was observed (Table 1). Also, there was a higher proportion of younger parents (p < 0.001) with higher educational status (p < 0.001) among children/adolescents characterized by a higher consumption of UPF (Table 1). A multivariate analysis for demographic variables showed that there was a significant association between being older and with obesity and high UPF consumption (OR = 2.74, 95% CI: 1.94, 3.88 for category 15–17 years, and OR = 1.97, 95% CI: 1.39, 2.80, respectively) and daily consumption of UPF (OR = 3.78, 95% CI: 2.12, 6.75 for category 15–17 years, and OR = 2.02, 95% CI: 1.09, 3.72, respectively) (Table 1). Similar findings were found for higher parental educational level, yet related only to high UPF consumption (OR = 2.23, 95% CI: 1.15, 4.31). On the contrary, in participants with questionnaires filled by a female parent and with older parents a significant inverse association with both high (OR = 0.66, 95% CI: 0.51, 0.85; OR = 0.20, 95% CI: 0.14, 0.29, respectively) and daily (OR = 0.47, 95% CI: 0.30, 0.73; OR = 0.38, 95% CI: 0.21, 0.71, respectively) consumption of UPFs was found (Table 1).

**TABLE 1:** Demographic characteristics of parents and children/adolescents participating in the DELICIOUS project according to the level of ultra-processed food consumption (Italy, Spain, Portugal, Egypt, and Lebanon. 2023–24).

	Consumption of UPF			High consumption of UPF	Daily consumption of UPF
	Low	High	P-value	OR (95% CI)^a^	OR (95% CI)^a^
Age groups, (n, %)			0.165		
6–8 years	251 (28.5)	294 (26.0)		1	1
9–11 years	230 (26.1)	272 (24.0)		1.68 (1.19, 2.36)	2.11 (1.27, 3.52)
12–14 years	205 (23.3)	273 (24.1)		2.01 (1.42, 2.85)	1.95 (1.18, 3.22)
15–17 years	194 (22.0)	292 (25.8)		2.74 (1.94, 3.88)	3.78 (2.12, 6.75)
Sex, (n, %)			0.064		
Male	456 (51.8)	539 (47.7)		1	1
Female	424 (48.2)	592 (52.3)		1.14 (0.90, 1.45)	1.38 (0.94, 2.03)
Weight status, (n, %)			<0.001		
Normal weight	551 (75.4)	536 (63.0)		1	1
Overweight	107 (14.6)	156 (18.3)		1.36 (0.97, 1.90)	1.55 (0.87, 2.77)
Obese	73 (10.0)	159 (18.7)		1.97 (1.39, 2.80)	2.02 (1.09, 3.72)
Parents age, (n, %)			<0.001		
<44 years	96 (10.9)	327 (28.9)		1	1
≥45 years	784 (89.1)	804 (71.1)		0.20 (0.14, 0.29)	0.38 (0.21, 0.71)
Parents occupational level, (n, %)			0.495		
Unemployed	678 (78.1)	855 (76.8)		1	1
Current working	190 (21.9)	258 (23.2)		1.20 (0.87, 1.66)	0.91 (0.55, 1.49)
Parents educational level, (n, %)			<0.001		
Low	56 (6.6)	35 (3.2)		1	1
Medium	399 (47.0)	351 (32.4)		1.57 (0.82, 3.02)	1.03 (0.34, 3.10)
High	394 (46.4)	699 (64.4)		2.23 (1.15, 4.31)	0.83 (0.28, 2.51)

^a^ Analyses were adjusted for all variables presented in the table.

The eating behaviours of participants according to the consumption of UPFs are presented in Table 2. There were significant differences in the consumption of UPFs, with higher rates of participants always eating breakfast, never/seldom eating out of home, daily eating with family, never/seldom eating alone and at school, not eating advertised food, and never/seldom eating snacks among lower UPF consumption group (p < 0.001, respectively for all variables, Table 2). Multivariate analysis showed that one or 2 or more times eating out of home was significantly associated with high (OR = 2.25, 95% CI: 1.80, 2.81; OR = 4.42, 95% CI: 2.88, 6.77, respectively) and daily (OR = 2.34, 95% CI: 1.61, 3.40; OR = 20.73, 95% CI: 2.85, 150.65; respectively) UPF consumption (Table 2). In line, eating advertised foods was significantly associated with both high (OR = 2.40, 95% CI: 1.93, 2.99) and daily (OR = 5.03, 95% CI: 3.24, 7.82) UPF consumption (Table 2). However, often eating snacks was also significantly associated with high UPF consumption (OR = 1.95, 95% CI: 1.02, 3.71). When considering often eating alone and often eating at school, a significant association was found only for high UPF consumption (OR = 1.81, 95% CI: 1.35, 2.43; OR = 1.47, 95% CI: 1.14, 1.91, respectively, Table 2), but not for daily UPF consumption.

**TABLE 2:** Eating behaviors of children/adolescents participating in the DELICIOUS project according to the level of ultra-processed food consumption (Italy, Spain, Portugal, Egypt, and Lebanon. 2023–24).

	Consumption of UPF			High consumption of UPF	Daily consumption of UPF
	Low	High	P-value	OR (95% CI)^a^	OR (95% CI)^a^
Breakfast habit, (n, %)			<0.001		
Never/seldom	80 (9.1)	197 (17.4)		1	1
Often	79 (9.0)	269 (23.8)		1.42 (0.94, 2.16)	1.18 (0.58, 2.40)
Always	721 (81.9)	665 (58.8)		0.90 (0.64, 1.28)	0.95 (0.54, 1.67)
Eating out of home, (n, %)			<0.001		
Never/seldom	592 (67.3)	340 (30.1)		1	1
Often/always	288 (32.7)	791 (69.9)		2.69 (2.18, 3.72)	1.74 (0.84, 3.61)
Eating with family, (n, %)			<0.001		
Seldom	16 (1.8)	25 (2.2)		1	1
Often	126 (14.3)	475 (42.0)		1.39 (0.66, 2.93)	2.27 (0.79, 6.50)
Daily	738 (83.9)	631 (55.8)		1.01 (0.48, 2.11)	1.81 (0.66, 5.01)
Eating alone, (n, %)			<0.001		
Never/seldom	693 (78.8)	554 (49.0)		1	1
Often	125 (14.2)	472 (41.7)		1.81 (1.35, 2.43)	1.17 (0.72, 1.90)
Daily	62 (7.0)	105 (9.3)		1.25 (0.85, 1.83)	1.22 (0.64, 2.32)
Eating at school, (n, %)			<0.001		
Never/seldom	438 (49.8)	393 (34.7)		1	1
Often	180 (20.5)	442 (39.1)		1.47 (1.14, 1.91)	1.31 (0.87, 1.98)
Almost daily	262 (29.8)	296 (26.2)		1.14 (0.90, 1.46)	0.93 (0.65, 1.32)
Eating advertised foods, (n, %)			<0.001		
No	631 (71.7)	400 (35.4)		1	1
Yes	249 (28.3)	731 (64.6)		2.40 (1.93, 2.99)	5.03 (3.24, 7.82)
Eating snacks, (n, %)			<0.001		
Never/seldom	158 (18.0)	94 (8.3)		1	1
Often/always	232 (26.4)	659 (58.3)		1.95 (1.02, 3.71)	2.88 (2.00, 4.16)

^a^ Analyses were adjusted for all variables presented in the table.

The distribution of lifestyle habits of children/adolescents according to the consumption of UPF is presented in Table 3. Among the low UPF consumption group, there were higher rates of participants reporting lower screen time (p < 0.001) and higher healthy lifestyle score (p < 0.001; Table 3). Spending >4 h/day or 2–4 h/day on screen was significantly associated with UPF consumption when considering both high (OR = 2.32, 95% CI: 1.59, 3.38 and OR = 2.14, 95% CI: 1.74, 2.63, respectively) and daily UPF (OR = 2.64, 95% CI: 1.31, 5.34 and OR = 2.61, 95% CI: 1.82, 3.76, respectively) consumption (Table 3). Conversely, there was a significant inverse association between medium and high healthy lifestyle score and high (OR = 0.33, 95% CI: 0.26, 0.42 and OR = 0.18, 95% CI: 0.14, 0.23, respectively) and daily (OR = 0.62, 95% CI: 0.41, 0.94 and OR = 0.34, 95% CI: 0.23, 0.49, respectively) UPF consumption (Table 3).

**TABLE 3:** Lifestyle habits of children/adolescents participating in the DELICIOUS project according to the level of ultra-processed food consumption (Italy, Spain, Portugal, Egypt, and Lebanon. 2023–24).

	Consumption of UPF			High consumption of UPF	Daily consumption of UPF
	Low	High	P-value	OR (95% CI)^a^	OR (95% CI)^a^
Sleep duration, (n, %)			0.025		
Less than 8 h	139 (15.8)	232 (20.5)		1	1
8–10 h	698 (79.3)	844 (74.6)		0.91 (0.71, 1.18)	1.04 (0.70, 1.56)
>10 h	43 (4.9)	55 (4.9)		0.81 (0.49, 1.33)	0.54 (0.28, 1.05)
Screen time, (n, %)			<0.001		
<2 h/day	597 (67.8)	534 (47.2)		1	1
2–4 h/day	234 (26.6)	489 (43.2)		2.14 (1.74, 2.63)	2.61 (1.82, 3.76)
>4 h/day	49 (5.6)	108 (9.5)		2.32 (1.59, 3.38)	2.64 (1.31, 5.34)
Physical activity level, (n, %)			0.047		
Low	470 (53.4)	547 (48.4)		1	1
Medium	182 (20.7)	279 (24.7)		1.27 (1,00, 1.63)	1.23 (0.83, 1.83)
High	228 (25.9)	305 (27.0)		1.19 (0.94, 1.50)	0.92 (0.65, 1.29)
Healthy lifestyle score^b^, (n, %)			<0.001		
Low	181 (20.6)	597 (52.8)		1	1
Medium	279 (31.7)	297 (26.3)		0.33 (0.26, 0.42)	0.62 (0.41, 0.94)
High	420 (47.7)	237 (21.0)		0.18 (0.14, 0.23)	0.34 (0.23, 0.49)

^a^ Analyses were adjusted for all variables presented in the table.

^b^ Referring to the lifestyle items of the E-KINDEX.

## Discussion

This research sought to explore the consumption of unhealthy UPFs among the younger populations in the Mediterranean region. The study specifically targeted children and adolescents from five countries: by examining dietary habits in these diverse areas, the study aimed to provide a comprehensive understanding of how unhealthy UPFs are consumed across different cultural and geographical contexts within the Mediterranean basin. The study found that a majority of children eat unhealthy UPF every day, raising concerns about public health. This issue is notably more severe among older children and those struggling with obesity.

Childhood obesity is alarming due to its potential long-term health effects, such as higher risks of heart disease, type-2 diabetes, and other chronic conditions [30]. Comprehensive overviews of the scientific literature suggest that UPF consumption is associated with higher BMI in both adults [31] and younger individuals [21]. With specific reference to some studies conducted in the Mediterranean region, a cross-sectional study conducted in Greece between 2014 and 2016 on a sample of 1,718 pre-adolescents, with an average age of approximately 11 years, revealed that those who were categorized as overweight or obese were also identified as high consumers of junk food [32]. Another study conducted in Portugal on 1175 children of the population-based birth cohort Generation XXI, with the aim to investigate UPF consumption, appetitive traits and BMI in children between the ages of 4 and 7, has demonstrated that the early consumption of UPF negatively impacts children’s BMI [33]. Although there is substantial research linking UPF consumption to health outcomes in adults, it is crucial to further explore its impact on children and adolescents [20]. Our study showed that adolescents are more likely to consume more unhealthy UPFs than children. This finding is consistent with other European studies on this matter: the study conducted within the Upper Project demonstrated that a higher percentage of older children from European countries was also allocated to a “sweet and processed” model, whereas a greater proportion of Spanish adolescents was observed in a “health-conscious” model [34]. The study also revealed that higher consumption of these foods is associated with higher parental education levels and younger age. This suggests that socioeconomic and demographic factors significantly influence children’s dietary choices [35], from the very first months of life [36]. In families with higher educational attainment, there may be greater exposure to nutritional information as well as increased financial means to purchase packaged and ready-to-eat foods [37]. Parents provide the food environments for their children’s early experiences with food and eating. These family food environments include the parents’ eating behaviors and their feeding practices with their children [38]. For example, the results of a research on the behavioral mediators of family patterns of overweight indicate that parents’ eating behaviors and their parenting practices influence the development of children’s eating behaviors, mediating family patterns of overweight. In particular, overweight parents who struggle to control their own food intake or are concerned about the risk of their children becoming overweight may adopt controlling feeding practices in an attempt to prevent overweight in their children [38].

Examining dietary habits, it was observed that children who eat out more frequently, such as at restaurants or fast food establishments, also tend to consume a greater amount of unhealthy UPFs. This behavior could be attributed to the greater availability and accessibility of these foods outside the home, and the fact that restaurants and food chains often offer large portions and calorie-dense foods high in fats and sugars, which can be very appealing to children [39]. Additionally, consuming more meals per day appears to be associated with increased UPF consumption, likely because more meals provide more opportunities to choose unhealthy foods [40]. In this regard, a study conducted using data from the Portuguese National Food, Nutrition and Physical Activity Survey (IAN-AF 2015/2016), which aimed to describe and compare energy intake, nutrient intake, and food consumption, showed that eating patterns in places other than home were strongly associated with higher energy intake and, in particular, restaurants and other locations, with poorer dietary adequacy [41]. This suggests that the external food environment and meal frequency can significantly influence children’s food choices, leading them to prefer less healthy options. For instance, a child who snacks frequently throughout the day may be more inclined to choose UPF snacks over fresh fruit or vegetables [42]. These eating patterns have undergone significant changes over the years. Indeed, regarding food availability, research conducted many years ago demonstrated that when young children were provided with a variety of healthy foods without adult intervention to control or influence their eating, they tended to choose diets that supported proper growth and health [43]. The key to the children’s success was the assortment of healthy, unseasoned, and unprocessed foods made available to them [44].

The overall diet quality of the study sample has been reported in recent study to be relatively good and in line with traditional Mediterranean pattern in a large share of participants [45, 46]. Although not significant in the multivariate analysis, there was a lower percentage of UPF consumption among children and adolescents eating with family compared with those eating alone. We hypothesize that higher diet quality may be the result of cultural habits rather than a direct health conscious choice. Restricting access to appealing snacks or “junk” food might seem like a simple method for teaching children moderation in consuming foods that are high in sugar, fat, and energy density but low in essential nutrients. However, studies indicate that well-meaning efforts by parents to help their children manage their food intake may inadvertently contribute to the development of potentially unhealthy eating habits [47, 48]. Utilizing both experimental and observational research methods, previous studies showed that limiting preschoolers’ access to specific foods increased their attention to and consumption of those restricted items when they became available, even if they had not been present previously. Similarly, girls’ perceptions of pressure and restriction were linked to the emergence of dietary restraint and disinhibition in these young girls [49].

Concerning lifestyle behaviors, the study found a strong link between increased screen time, such as time spent watching TV, using computers, and mobile devices, and higher consumption of unhealthy UPF. This connection may stem from the fact that children who see advertisements for unhealthy foods, which are often heavily promoted through media and TV commercials, might be more inclined to crave and consume these products [50]. These ads frequently use bright colors, cartoon characters, and catchy phrases to attract children to consume food that, however, is not nutritionally healthy or suitable for the needs of this age group [51]. The aforementioned study conducted on 1,718 pre-adolescents in Greece, regarding this aspect, demonstrated that the television environment, and particularly the advertising environment, has a significant and negative impact on the eating habits of pre-adolescents. Specifically, those who claim that their lifestyle and food choices are mainly influenced by advertising are 45% more likely to be heavy consumers of junk food [32]. Additionally, excessive screen time may limit opportunities for engaging in healthy physical activities, further contributing to an overall less healthy lifestyle [52]. A study conducted in Portugal aimed at investigating UPF consumption and its association with risk of obesity and sedentary behaviors in Portuguese adolescents demonstrated that adolescents who consumed more ultra-processed foods also had a sedentary lifestyle [53]. Another study carried out in several European countries, including Spain and Greece, examined the connection between food and beverage consumption and television viewing habits among adolescents. The study, known as the Healthy Lifestyle in Europe by Nutrition in Adolescence (HELENA), revealed that spending excessive time watching TV may lead to an increased likelihood of consuming high-calorie snacks and sugary beverages simultaneously [54]. This behavior highlights a potential link between screen time and unhealthy dietary habits in adolescents [55]. Similarly, an overall unhealthier lifestyle (evaluated with the E-KINDEX, which includes factors like regular physical activity, good sleep quality, and effective stress management) is associated with higher intake of UPF. This indicates that children with generally poorer health habits are also more likely to make less nutritious food choices. In line with this, adolescents also have poor adherence to the Mediterranean diet, as demonstrated by a study conducted in Morocco that investigated adherence to the Mediterranean diet among high school students, revealing a low level of adherence [56]. In other words, there is a noticeable correlation between a generally unhealthy lifestyle and a diet high in UPF. A higher consumption of UPF was also associated with lower diet quality in the Italian “I.Family” study, which aimed to investigate the association between UPF intake and the nutritional quality of the diet among European children and adolescents [57].

### Limitations

To the best of our knowledge, this study has the strength to evaluate the association between the consumption of unhealthy UPFs and the behavioral patterns of children and adolescents in five Mediterranean countries using a common methodology. However, the conclusions of this study should be considered in light of some limitations. The cross-sectional design of the study does not allow for causal associations to be drawn. Another limitation could be the reporting bias due to the questionnaires completed by the parents of the children regarding dietary frequencies and eating habits. Furthermore, the amount of UPF derived from the FFQ may not provide an accurate estimate of the quantity consumed. Additionally, since this study only focuses on junk foods, the level of processing is considered a proxy for unhealthy formulations that could, in fact, affect detrimental health outcomes.

### Conclusion

In conclusion, the consumption of unhealthy UPF is notably high among children and adolescents living in the Mediterranean region. A range of factors, including the child’s age, obesity status, parental education, eating habits, and overall lifestyle play a significant role in influencing how much unhealthy UPF children consume on a daily basis. All such factors represent a lifestyle pattern that is hard to disentangle, making it nearly impossible (with the available data) to establish direct connections with individual variables. This situation underscores the necessity for a well-rounded approach that considers both dietary choices and lifestyle behaviors in order to foster healthier eating patterns among children. Addressing these factors comprehensively is crucial for promoting better nutrition and overall health. To achieve this, it is imperative that parents, educators, and healthcare professionals collaborate closely. By working together, they can help create a supportive environment that encourages nutritious food choices and an active lifestyle. Such an environment should provide resources, education, and practical strategies that enable children and their families to make healthier decisions. This collective effort aims to not only improve dietary habits but also enhance physical activity, ultimately contributing to better health outcomes and wellbeing for children.
